# *Tachypleus tridentatus* Lectin Enhances Oncolytic Vaccinia Virus Replication to Suppress In Vivo Hepatocellular Carcinoma Growth

**DOI:** 10.3390/md16060200

**Published:** 2018-06-07

**Authors:** Gongchu Li, Jianhong Cheng, Shengsheng Mei, Tao Wu, Ting Ye

**Affiliations:** College of Life Sciences, Zhejiang Sci-Tech University, Hangzhou 310018, China; cjh15067149909@163.com (J.C.); mss1053280369@163.com (S.M.); wutao0920@163.com (T.W.)

**Keywords:** TTL, oncolytic vaccinia virus, viral replication, ERK

## Abstract

Lectins play diverse roles in physiological processes as biological recognition molecules. In this report, a gene encoding *Tachypleus tridentatus* Lectin (TTL) was inserted into an oncolytic vaccinia virus (oncoVV) vector to form oncoVV-TTL, which showed significant antitumor activity in a hepatocellular carcinoma mouse model. Furthermore, TTL enhanced oncoVV replication through suppressing antiviral factors expression such as interferon-inducible protein 16 (IFI16), mitochondrial antiviral signaling protein (MAVS) and interferon-beta (IFN-β). Further investigations revealed that oncoVV-TTL replication was highly dependent on ERK activity. This study might provide insights into a novel way of the utilization of TTL in oncolytic viral therapies.

## 1. Introduction

The horseshoe crab, as a “living fossil”, has survived for more than 500 million years [1]. It can live solely on its hemolymph that contains granular hemocytes comprising 99% of the total hemocytes. The granules store many soluble defense molecules, such as lectins, clotting factors, clottable protein coagulogens, and C-reactive proteins [2,3]. Among those, Lectins, which are multivalent carbohydrate-binding proteins recognizing and binding with conserved pathogen associated molecular patterns (PAMPs) [4,5], agglutinate Gram-negative and Gram-positive bacteria by recognizing the structures of lipopolysaccharide (LPS) and lipoteichoic acid (LTA).

Lectins play diverse roles in physiological processes [6,7], including mediating interactions between cells during development and differentiation [8], and recognizing foreign molecules during immune responses [9]. Several lectins with a broad range of specificity have been identified in horseshoe crab [10]. In the Japanese horseshoe crab, there are six types of lectins, Tachylectin-1 (TL-1), Tachylectin-2 (TL-2), Tachylectin-3 (TL-3), Tachylectin-4 (TL-4) from hemocytes, and Tachylectin-5A (TL-5A) and Tachylectin-5B (TL-5B) from plasma. In the Taiwanese horseshoe crab, two types of lectins, *Tachypleus* plasma lectin 1 (TPL1) and *Tachypleus* plasma lectin 2 (TPL2), have been isolated and characterized as novel hemolymph proteins in the plasma of *Tachypleus tridentatus* [11]. TPL2, also named as *Tachypleus tridentatus* lectin (TTL), shows an 80% sequence identity with TL-3, and both TTL and TL-3 show ligand specificity toward lipopolysaccharides (LPSs), particularly O-antigen [12]. It has been reported that TTL directly interacted with L-rhamnose but not interact with D-galactose as demonstrated by a glycan array and Magnetic Reduction (MR) assay, implying that TTL might perform biological functions through recognizing rhamnose-containing molecules [12,13]. 

Oncolytic viruses are therapeutically useful viruses that preferentially replicate in cancer cells to elicit the killing effect [14]. A number of viruses including adenovirus, coxackie virus, vesicular stomatitis virus, measles virus, newcastle disease virus, parvovirus, poliovirus, reovirus, and vaccinia virus have now been clinically tested as oncolytic agents [15,16,17,18]. Vaccinia virus (VV) became famous as the most successful live biotherapeutic agent in the worldwide smallpox eradication program. VV replication occurs in the cytosol independent from the host cell nucleus [19,20]. Therefore, there is no possibility of chromosomal integration in contrast to other vector systems. Due to its unique features, VV is exploited as a therapeutic agent for the treatment of cancer. VV can be used for cancer therapy as cancer vaccines to stimulate antitumor immunity, or as a replicating virus vector sometimes harboring therapeutic genes to directly lyse tumor cells [21]. Arakawa et al. used an attenuated vaccinia virus to cure patients with metastatic lung and kidney cancer [22]. Kawa and Arakawa treated a multiple myeloma patient with the same attenuated vaccinia virus strain [23]. These studies indicated that oncolytic vaccinia virus had significant anticancer efficacy in various types of cancer. In previous studies, *Haliotis discus discus* sialic acid binding lectin (HddSBL), *Dicentrarchus labrax* fucose-binding lectin (DlFBL), and *Strongylocentrotus purpuratus* rhamnose-binding lectin (SpRBL) exogenously expressed through adenovirus vector showed suppressive effect on a variety of cancer cells in vitro [24,25]. Lectin from *Mytilus galloprovincialis* (MytiLec) was shown to be cytotoxic to diverse cancer cells through eliciting autophagy or apoptosis [26,27,28]. These data suggested that marine lectins may provide a distinct source of cancer therapeutic agents. Furthermore, our previous studies showed that oncolytic adenovirus vector harboring mannose binding lectin *Pinellia pedatisecta* agglutinin (PPA) showed an antileukemia effect in a mouse model [29], suggesting that harboring lectin genes may enhance the therapeutic effect of oncolytic viruses. In this study, marine lectin TTL was inserted to an oncolytic vaccinia virus (oncoVV) vector, which is deficient of thymidine kinase for cancer specific replication [30], to generate recombinant virus oncoVV-TTL. The antitumor effect of oncoVV-TTL and the underlying mechanisms were analyzed.

## 2. Results

### 2.1. oncoVV-TTL Suppressed Liver Cancer Cell Growth In Vivo

**Figure 1:**
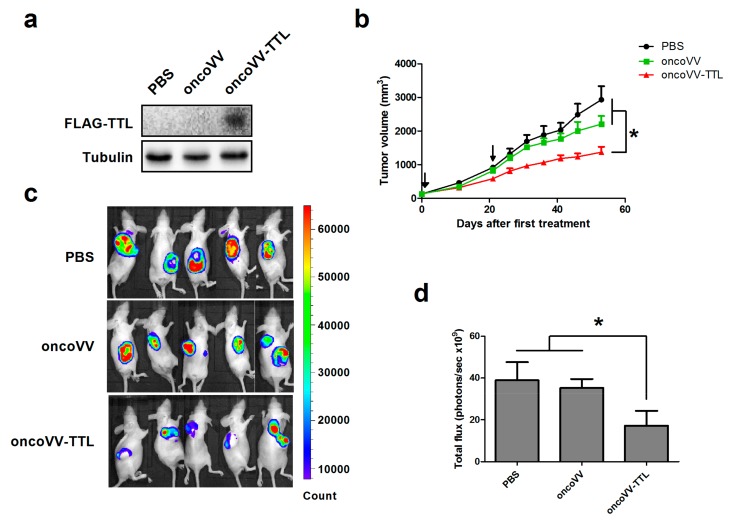
Intracellular expression of *Tachypleus tridentatus* lectin (TTL) in oncoVV-TTL infected cancer cells and the efficacy of oncoVV-TTL against hepatocellular carcinoma in vivo. (**a**) The expression of FLAG tagged TTL was determined by Western blot with an antibody against FLAG. Tubulin served as the loading control. (**b**) MHCC97-H cells were injected into the Balb/c nude mice. Mice were injected with PBS, oncoVV, or oncoVV-TTL after tumor size reached 120 mm^3^. Arrows indicate two injections. Values are displayed as mean tumor size ±SEM. Statistically significant differences between treatments were represented by asterisks (* *p* < 0.05). Tumor growth curve of MHCC97-H tumors treated by different injections. (**c**) Representative MHCC97-H tumors 44 days after first treatment. Mice were imaged using the IVIS imaging system. (**d**) Quantification of fluorescence intensity of the MHCC97-H tumors 44 days after first treatment.

The FLAG tagged TTL was detected through Western blot with an antibody against FLAG in oncoVV-TTL treated cancer cells, but not in cells treated with PBS or oncoVV (Figure 1a), indicating that TTL is able to be expressed in cancer cells. To assess the efficacy of oncoVV-TTL against liver cancer in vivo, Balb/c nude mice were subcutaneously engrafted with MHCC97-H liver cancer cells stably expressing fire fly luciferase to establish a tumor-bearing mouse model [31]. The mice then received two injections of oncoVV-TTL or oncoVV for 1 × 10^7^ plaque forming unit (PFU) each. PBS served as the negative control. As shown in Figure 1b, both oncoVV-TTL and oncoVV elicited antitumor efficacy. However, treatment with oncoVV-TTL resulted in a superior antitumor efficacy as compared to both oncoVV and PBS controls. Furthermore, bioluminescence was also monitored for the cancer cell burden in mice. Results confirm the antitumor effect of oncoVV-TTL as compared to oncoVV and PBS (Figure 1c). The significant suppressive effect of oncoVV-TTL compared with PBS and oncoVV was determined by statistical analysis (Figure 1d). Our data demonstrated the antitumor efficacy of oncoVV-TTL.

### 2.2. Oncolytic Vaccinia Virus Replication Improved by TTL

**Figure 2:**
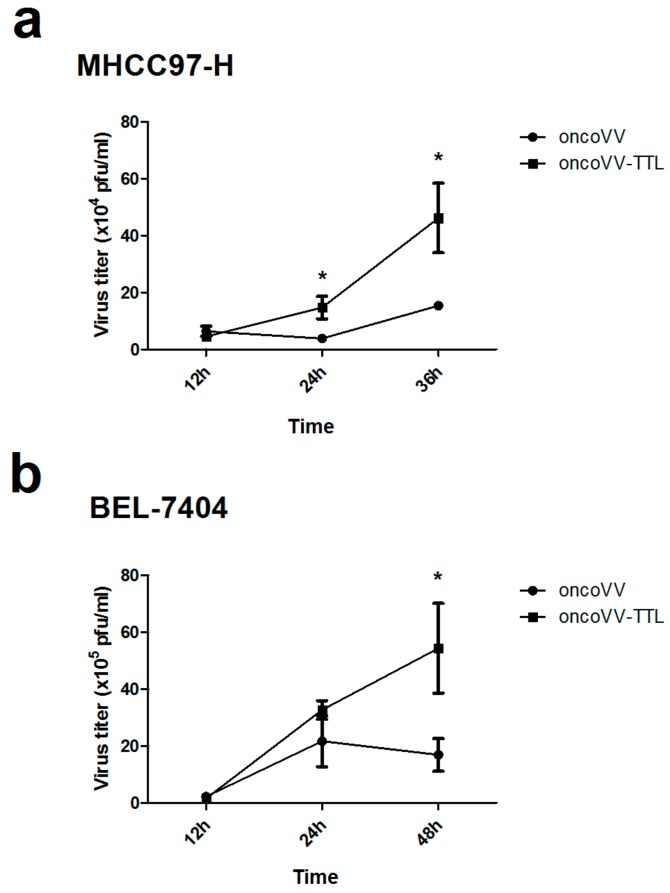
Replication of oncoVV-TTL in hepatocellular carcinoma cell lines. Replication of oncoVV and oncoVV-TTL in MHCC97-H cells (**a**) and BEL-7404 cells (**b**). Mean viral replication was determined by TCID_50_ assay on MHCC97-H cells. Statistical analysis was carried out using a Students unpaired *t* test at each time point. (* *p* < 0.05).

We then investigated the underlying mechanism of the antitumor effect of oncoVV-TTL. The viral replication was examined for oncoVV and oncoVV-TTL in liver cancer cell lines MHCC97-H and BEL-7404. As shown, oncoVV-TTL replicated significantly faster than oncoVV in MHCC97-H (Figure 2a), which was further confirmed in BEL-7404 cell line (Figure 2b). Thus, our data demonstrated that arming oncolytic vaccinia virus with TTL improved viral replication.

**Figure 3:**
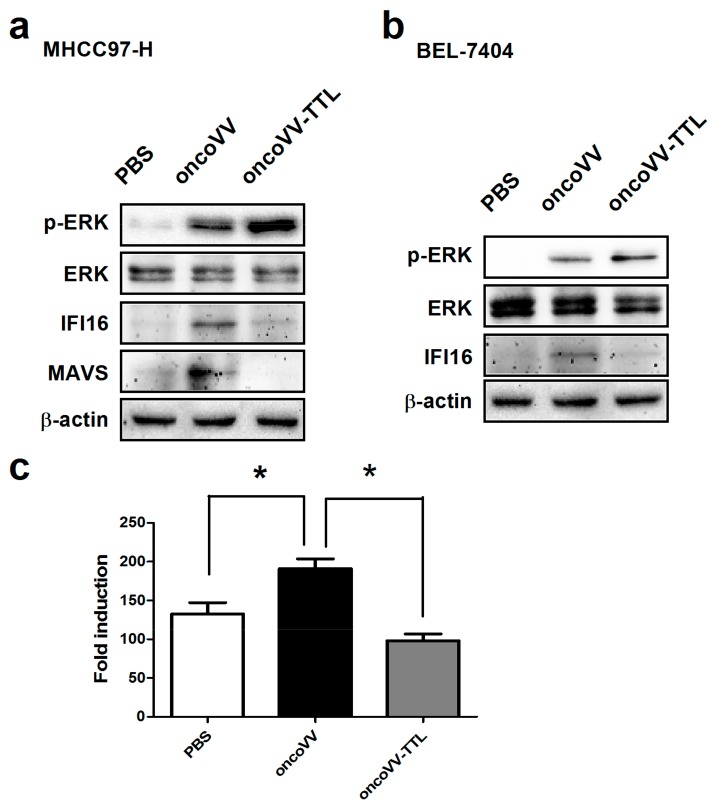
Intracellular signaling elements regulated by oncoVV-TTL. MHCC97-H cells (**a**) or BEL-7404 cells (**b**) were treated with 5MOI of oncoVV-TTL or oncoVV for 24 h, and cells were also treated with PBS as a negative control. Western blot was performed with antibodies against phosphor-extracellular signal-regulated kinase (ERK), ERK, interferon-inducible protein 16 (IFI16), mitochondrial antiviral signaling protein (MAVS) and β-actin. β-actin served as the loading control. (**c**) Activity of interferon-beta (IFN-β) promoter was analyzed through a duo-luciferase reporter assay kit. Statistically significant differences between treatments were represented by asterisks (* *p* < 0.05).

Intracellular signaling elements related to viral infection and replication were then analyzed. As reported previously, extracellular signal-regulated kinase (ERK) is required for vaccinia virus replication [32,33]. Interferon-inducible protein 16 (IFI16) senses viral DNA in the cytoplasm as well as the nucleus to initiate innate immune responses [34], and plays an important role in the initial steps of the inflammatory processes that precede the onset of autoimmune syndromes [35]. Mitochondrial antiviral signaling protein (MAVS) acts as an important factor in the induction of antiviral and inflammatory responses [36,37]. We then analyzed the phosphorylation level of ERK as well as the expression of IFI16 and MAVS through Western blotting. As shown in Figure 3a,b, oncoVV infection induced ERK phosphorylation in both MHCC97-H and BEL-7404 cell lines. Interestingly, oncoVV-TTL led to a significantly higher level of ERK phosphorylation as compared to oncoVV. We then investigated the effect of oncoVV-TTL on cellular levels of MAVS and IFI16. In MHCC97-H cells, oncoVV but not oncoVV-TTL induced the expression of MAVS and IFI16 (Figure 3a). In BEL-7404 cells, oncoVV triggered the expression of IFI16. On the contrary, oncoVV-TTL did not induce the IFI16 expression (Figure 3b). Our data demonstrated that TTL facilitated vaccinia virus replication in cancer cells through regulating intracellular signaling elements related to viral infection and replication.

Interferon-beta (IFN-β) regulates a wide range of genes, most of which are involved in the antiviral immune response, playing an important role in inducing non-specific resistance against a broad range of viral infections [38,39]. To determine the effect of oncoVV-TTL infection on IFN-β induction, IFN-β reporter assay was performed in MHCC97-H cells. Results showed that oncoVV induced the upregulation of IFN-β transcription, which was significantly suppressed through TTL harboring (Figure 3c). Taken together, our results indicated that TTL favors oncolytic vaccinia virus replication through suppressing the antiviral response of cancer cells.

### 2.3. The Role of ERK Activity on oncoVV-TTL Replication

**Figure 4:**
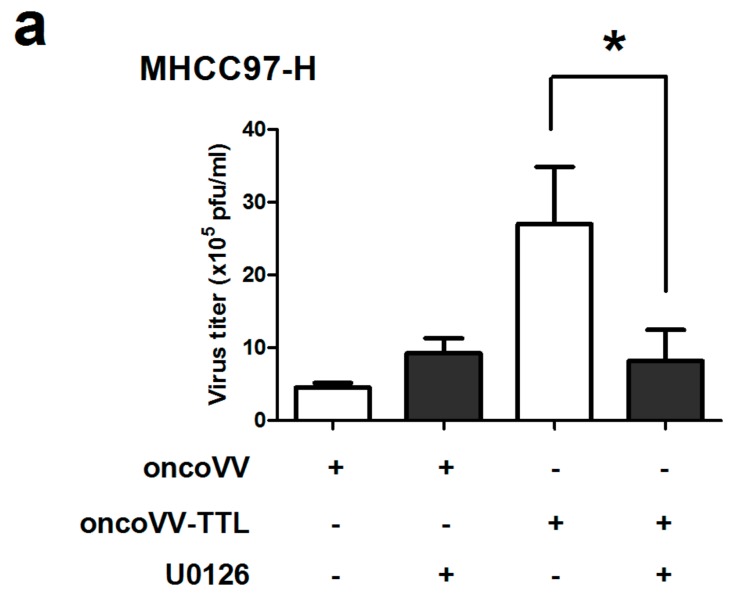

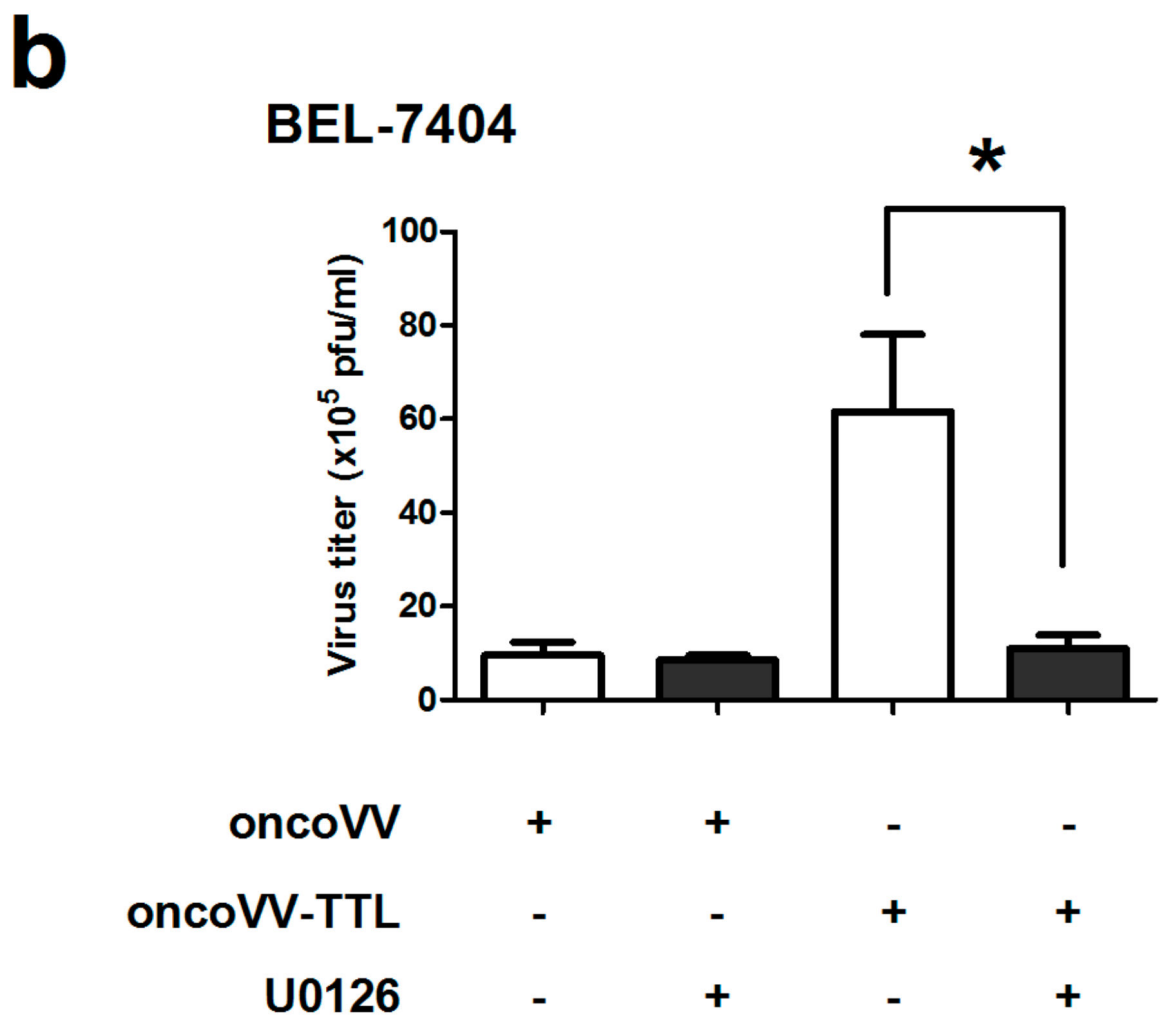
Virus replication was dependent on ERK activity. MHCC97-H cells (**a**) or in BEL-7404 cells (**b**) were infected with oncoVV, oncoVV-TTL respectively with or without the combination of ERK inhibitor U0126. Virus titers were then measured through TCID_50_ aasay. Statistically significant differences between treatments were represented by asterisks (* *p* < 0.05).

The role of ERK activity in oncoVV-TTL replication was further analyzed. U0126, an inhibitor of mitogen-activated protein kinase kinase (MEK) 1/2-mediated phosphorylation of ERK1/2 [40,41], was used to treat liver cancer cell lines in combination with oncoVV or oncoVV-TTL. Results showed that in MHCC97-H cells the virus titers of oncoVV-TTL but not oncoVV were markedly reduced with the combination of U0126 (Figure 4a). In BEL-7404 cell lines, the effect of U0126 on oncoVV-TTL and oncoVV replication yielded essentially similar results as in MHCC97-H (Figure 4b). Our results indicated that oncoVV-TTL replication was highly dependent on ERK activity.

## 3. Discussion

The utilization of lectins in antitumor therapies are greatly limited by their in vivo immunogenicity and toxicity. Vaccinia viruses provide promising vectors for oncolytic therapies and have been developed to be valuable agents for preclinical and clinical evaluations due to their safety and effect [42,43,44,45]. In the work presented, A *Tachypleus tridentatus* plasma lectin TTL was genetically inserted into an oncoVV vector and the antitumor activity was evaluated. We showed that TTL enhanced the antitumor activity of oncoVV due to its ability to promote virus replication in liver cancer cells. Further studies showed that the TTL harboring significantly suppressed the oncoVV induced antiviral factors, and the replication of oncoVV-TTL was highly dependent on ERK activation. Importantly, our study did not find obvious toxicity of oncoVV-TTL in this hepatocellular carcinoma mouse model. Therefore, our studies suggest that harboring lectin genes in oncolytic viral vectors could be an important novel direction to overcome the in vivo toxicity of lectins for further development of lectin based antitumor agents. 

Viruses need to overcome host antiviral responses for effective replication and spreading. Human cells have evolved a series of viral restriction factors that directly inhibit various steps of viral replication [46,47]. Nucleus associated IFI16 protein, as an innate DNA sensor, regulates inflammatory cytokines and type I interferon (IFN) production [48]. In addition, Mitochondrial antiviral signaling protein (MAVS) acts as an important factor in the induction of antiviral and inflammatory responses [49]. In this study, TTL upregulated the oncoVV induced ERK phosphorylation and suppressed the antiviral factors such as IFN-β, IFI16 and MAVS induced by oncoVV. Therefore, the relationship between ERK activity and antiviral factors still remains unclear pending further investigations.

## 4. Materials and Methods

### 4.1. Cell Culture and Transfection

The human embryonic kidney cell line 293A, hepatocellular carcinoma cell lines MHCC97-H and BEL-7404 were provided by American Type Culture Collection (Rockville, MD, USA). Cells were incubated in Dulbecco’s modified Eagle’s medium supplemented with 1% penicillin/streptomycin solution, 1% L-Glutamine and 10% fetal bovine serum, maintained at 37 °C in a humidified 5% CO_2_. Appropriate amounts of plasmids were transfected into cells by using Thermo Scientific TurboFect Transfection Reagent (Thermo Fisher Scientific Inc., Waltham, MA, USA) following the manufacturer’s instruction.

### 4.2. Plasmid Construction

The plasmid pEGFP-Flag-TTL encoding TTL (GenBank accession no. AF264068) gene was purchased from Shanghai Generay Biotech Co., Ltd., Shanghai, China. For recombinant expression in cell lines MHCC97-H and BEL-7404, the Flag-TTL gene was cloned into the pCB plasmid using primers 5′-GA*AGATCT*ATGGATTACAAGGATGACGACGATAAGGGAATTTTCAAAGTGT-3′ (forward) with a *Bgl*II site (italic) and 5′-GC*TCTAGA*TTACTTAATTATTATAATAGGTCCA-3′ (reverse) with a *Xba*I site (italic). The sequence was confirmed by Shanghai Generay Biotech Company.

### 4.3. Vaccinia Virus Construction

The vaccinia virus was generated in our laboratory previously. After HEK-293A cells infected with wild type vaccinia virus about 2–4 h, pCB-Flag-TTL were transfected into 293A cells. Mycophenolic acid, dioxopurine, and hypoxanthine were added to screen effective oncoVV-TTL. Recombinant viruses were gathered from cell culture medium, and purified through CsCl gradient centrifugation. The virus titers were determined by TCID_50_ (median tissue culture infective dose).

### 4.4. Infectious Progeny Production

To determine virus progeny production, 5× 10^4^ cells (MHCC97-H, BEL-7404) were plated in 24-well plates. Cells were infected with 5MOI (multiplicity of infection) of the Vaccinia virus oncoVV-TTL or oncoVV. After 24 h, 36 h and 48 h, cells were collected and washed twice with PBS. After three freeze/thaw cycles in −80 °C and 37 °C, the production was determined by TCID_50_ assay on 293A cells.

### 4.5. Animal Experiments

Balb/c nude mice of 4–5 weeks age were used for hepatocellular carcinoma tumor-bearing mouse model. MHCC97-H at 2.5 × 10^6^ cells/mouse were injected subcutaneously into the mice on the back. Mice were randomly grouped and in situ injected with two injections of oncolytic vaccinia virus for 1 × 10^7^ plaque-forming units (PFU) each. Then we measured the volume of tumor every five days. The tumor volume was calculated using the formula: length (mm) × width (mm)^2^ × 0.5. After injection of D-luciferin into mice, bioluminescence was measured through the Caliber IVIS kinetics (Caliper life sciences, Hopkinton, MA, USA). Regions of interest were measured through the IVIS software.

All animal studies were approved by the Institutional Animal Care and Use Committee (IACUC) of Zhejiang Sci-Tech University (2017-1), Hangzhou, Zhejiang, China.

### 4.6. Reporter Assay

IFN-β firefly luciferase reporter plasmid was constructed previously. Reporter assay was performed using a duo-luciferase assay kit (GeneCopoeia, Inc., Rockville, MD, USA) according to the manufacturer’s instructions. Briefly, MHCC97-H or BEL-7404 cells were co-transfected with Renilla luciferase control plasmid and IFN-β luciferase reporter plasmid, followed by treatment with PBS, 5 MOI of oncoVV or oncoVV-TTL for 24 h. Then cells were lysed and IFN-β luciferase activity was normalized to Renilla luciferase activity.

### 4.7. Western Blotting Analysis

The cell extracts were separated in SDS-PAGE gel and transferred onto nitrocellulose membranes. The membranes were then immersed in Tris-buffered saline and Tween-20 containing 5% of bovine serum albumin at room temperature for 1 h. Subsequently, the membrane was incubated with the primary antibody, followed by incubation with secondary antibodies for 1 h at room temperature. After washing with Tris-buffered saline, the bands were detected under a Tanon 5500 chemiluminescence image system (Tanon Inc., Shanghai, China).

Goat anti-MAVS, IFI16 antibodies were purchassed from Santa Cruz Biotechnology Inc. (Dallas, TX, USA). Rabbit anti-ERK1/2 and phospho-ERK1/2 antibodies were purchased from Cell Signaling Technology Inc. (Danvers, MA, USA). Rabbit anti-β-actin was purchased from Bioss Antibodies (Beijing, China). The HRP conjugated goat anti-rabbit and goat anti-mouse antibodies were purchased from MultiSciences (Lianke) Biotech Co., Ltd. (Hangzhou, China).

### 4.8. Statistical Analysis

Differences among the different treatment groups were determined by student’s *t*-test. *p* < 0.05 was considered significant. 

## 5. Conclusions

Our studies showed that oncoVV-TTL elicited significant antitumor activity in a hepatocellular carcinoma mouse model. TTL enhanced viral replication through inhibiting the antiviral immune response in hepatocellular carcinoma cells. Furthermore, oncoVV-TTL replication was demonstrated to be depended on ERK activity. Our studies might provide insights into the utilization of marine lectin genes such as TTL in oncolytic viral therapies. However, the underlying mechanism of the TTL functions in cancer cells still remains unclear pending further investigations.
